# Clinical-experimental assessment of simvastatin efficiency in the treatment of rheumatoid arthritis

**DOI:** 10.1186/ar3666

**Published:** 2012-02-09

**Authors:** Rikhikhon N Tadjikhodjaeva, Nargiza G Khabibullaeva

**Affiliations:** 1 Tashkent Medical Academy, Tashkent, Uzbekistan

## Subject of the inquiry

35 patients with rheumatoid arthritis, 50 mature male rats of mixed population.

## Aim of the inquiry

Clinical-experimental assessment of simvastatin efficiency and pathogenic justification of its inclusion into the complex treatment for therapy optimization in patients with rheumatoid arthritis.

## Methods of investigation

clinical-laboratory, biochemical - determination of total cholesterol, low and high density lipoproteins, triglycerides, calculation of atherogenic coefficient in blood serum of patients with rheumatoid arthritis and in experimental animals.

## The results achieved and their novelty

On the systemic and local levels an approach was applied allowing consideration of nitrogen oxide metabolism disorders as an important part of the pathogenesis of rheumatoid arthritis. A number of new data were obtained concerning the relationship of nitrogen oxide metabolism and C-reactive protein formation, clinical course of rheumatoid arthritis. For the first time a complex approach was suggested for the pathogenic justification of simvastatin use in the scheme of conventional treatment to increase the therapy efficiency, to achieve stable early remission in patients with rheumatoid arthritis. It was proved that an important mechanism of increasing the therapeutic efficiency of simvastatin was its action on the system of endothelial function in blood and joint fluid. It was suggested that one should include assessment of blood and joint fluid for nitrogen oxide, nitrate diaphorase and nitrate reductase in the algorithm of investigation and dynamic observation, choice of tactics and therapy efficiency assessment.

## Practical value

Obtained new data are necessary for increasing the pharmacotherapy efficacy in patients with rheumatoid arthritis taking into account the metabolic activity of NO-synthetase mechanism in blood and synovial fluid. An algorithm was suggested for screening observation and differentiated management of patients with rheumatoid arthritis taking account of severity of nitrogen oxide metabolism disorders. A differentiated approach was worked out and justified of simvastatin prescription both to increase the efficacy of treatment taking into account the clinical activity of the disease and to correct metabolic disorders in patients with rheumatoid arthritis.

